# Checkpoint inhibitors are a basic science‐based, transformative new treatment for lung cancer

**DOI:** 10.1111/resp.14437

**Published:** 2022-12-19

**Authors:** Alistair R. Miller

**Affiliations:** ^1^ Department of Respiratory and Sleep Medicine Royal Melbourne Hospital Parkville Victoria Australia; ^2^ Department of Internal Medicine Peter MacCallum Cancer Centre Parkville Victoria Australia; ^3^ Department of Medicine (RMH), Faculty of Medicine Dentistry and Health Sciences The University of Melbourne Parkville Victoria Australia

**Keywords:** animal models, basic science, checkpoint inhibitors, T cell biology

While refinements in existing therapies have led to small incremental improvement in lung cancer outcomes over the years, nothing has had quite the same impact as the development of immunotherapy, specifically checkpoint inhibitors. Immune checkpoint inhibitors first made headlines in the modern era following the 2010 publishing of a randomized controlled trial showing a significant survival benefit of ipilimumab in metastatic melanoma.^1^ Given the previously poor outcomes in advanced NSCLC,^2^ it is unsurprising that trials in NSCLC followed, again showing remarkable extended survival for some people.^3^, ^4^ In today's lung cancer treatment, checkpoint inhibitors form an essential part of the armoury, with progressive movement into earlier stage disease.^5^, ^6^ This commentary will emphasize the basic science behind checkpoint inhibitors to highlight recent insights into mechanism of action and possible complications.

**FIGURE 1:**
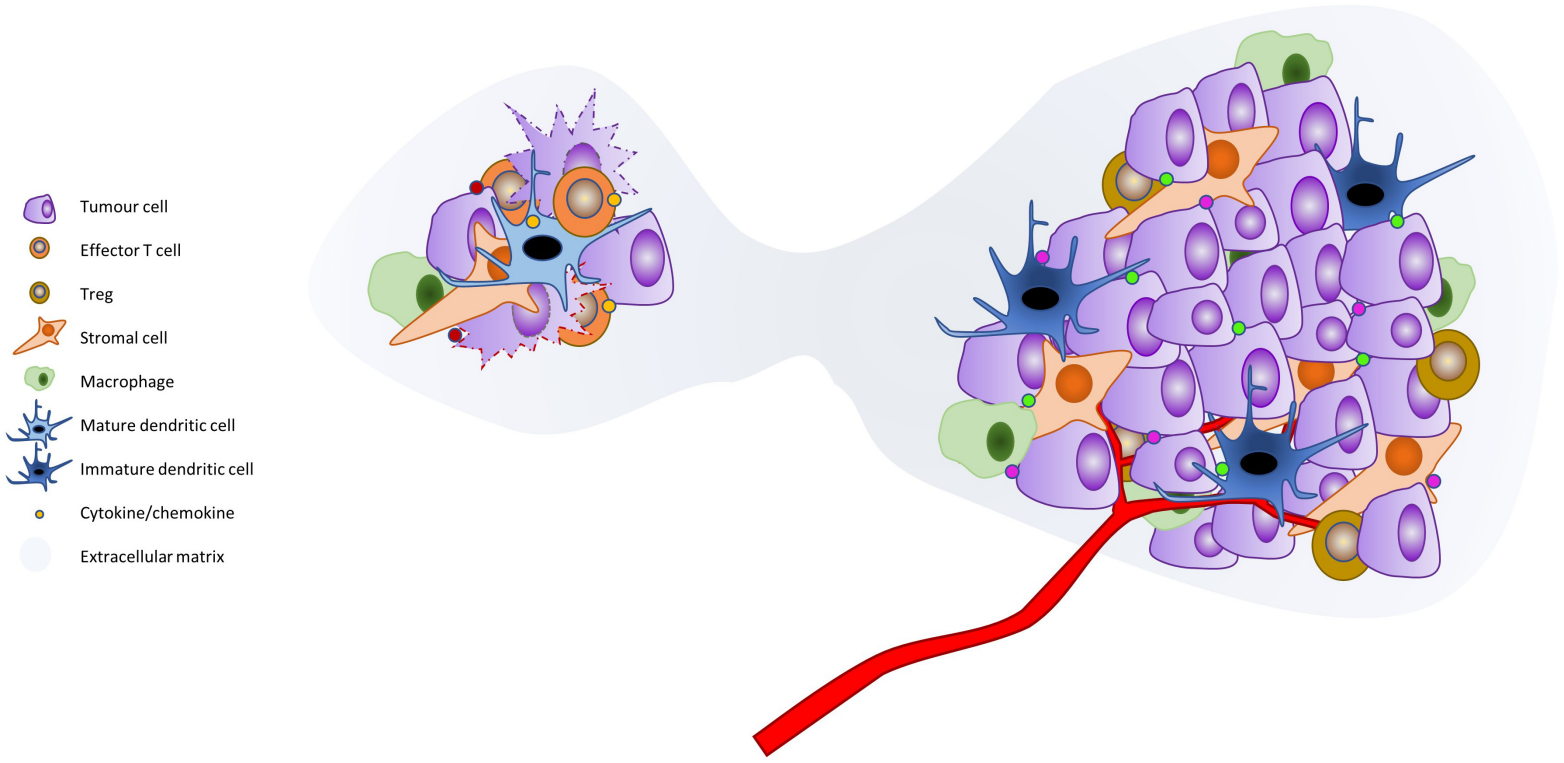
While cells transformation through transcriptional errors is frequent, mature dendritic cells and competent effector CD8+ T cells, under the influence of pro‐inflammatory cytokines, are able to recognize the non‐self cells and remove them. However, when tumour cells are able to develop a conducive microenvironment, they are able to switch the immune response to one of tolerance, enabling rapid cell growth in the context of immune evasion. Based on data from References 7, 62, 63.

The tumour microenvironment (TME) describes the interplay between tumour cells, infiltrating and adjacent immune cells, stromal cells, cellular components and non‐cell factors including extra‐cellular matrix (ECM), cytokines and chemokines^7^, ^8^ (Figure 1). With the tumour cell as the controlling central cell, the TME is essential at all points of tumour initiation, progression and metastasis. While not a new concept,^9^ the importance of this complex environment is increasingly evident in the era of immunotherapy. Since Virchow in the late 19th century,^10^ inflammation has been associated with cancer development, but the work of Hanahan and Weinberg formalized it, in their discussions of the ‘Hallmarks’ of cancer, as an enabling characteristic.^11^ While immune cell infiltrates seen on pathological sections were long thought to be indicative of anti‐tumour actions, there has been conflicting evidence of their prognostic significance.^12^ It is clear that new malignancies are more common in the context of both immunosuppression^13^ and dysregulated inflammation,^14^, ^15^, ^16^ but it has become increasingly evident that it is the type and characteristics of the inflammatory cells and molecules, rather than simply the presence of inflammation, that is important. While a spectrum of immune cells, including macrophages and myeloid derived suppressor cells, often traffic to the site of tumours and are thought to play a role in the overall microenvironment, it is largely dendritic cells (DCs) and lymphocytes that are key in response to immunotherapy.

Secretion of chemokines by tumour cells lead to recruitment of specific cell types and differentiation into cells conducive to immune evasion and tumour progression.^17^ Antigen presenting cells (APCs), and particularly DCs, are essential in immune surveillance and regulating the balance between immune tolerance of self, and active immune response to non‐self.^18^ While transcriptional errors frequently result in transformed cells,^19^ local mature DCs play a crucial role in presentation of non‐self‐tumour‐associated antigens (TAAs),^18^ targeting these cells for elimination. The presence of the TAA on major histocompatibility (MHC) class I molecules, along with appropriate co‐stimulatory molecules primes naïve T cells to mount a response against the non‐self threat.^20^ Indeed, the presence of tumour‐infiltrating mature DCs has been associated with a better prognosis in lung cancer.^21^ Conversely, if immature DCs lacking appropriate co‐stimulatory molecules are present, rather than leading to T cell activation and tumour cell destruction, they foster a tolerogenic environment.^22^ Immature DCs are constantly sampling the local environment for antigens, and while the detection of a TAA would under normal circumstances lead to maturation and migration to lymphoid tissue for presentation to T cells, local tumour factors including the presence of IL‐10 and VEGF can prevent this maturation.^18^, ^23^, ^24^ Tumours can actively suppress the maturation of DCs to aid in failure of immunosurveillance.^18^

Tumour‐infiltrating lymphocytes and macrophages are also seconded to promotion of tumour survival. DCs express several immunomodulatory molecules and in to responding to them, also secrete immune suppressing cytokines including IL‐10. This immunosuppressive milieu, along with down regulation of cell attachment molecules on tumour associated vessels,^25^ reduces the influx of tumour‐infiltrating lymphocytes that would otherwise form the effector arm of an anti‐tumour response.^26^ Those lymphocytes that do infiltrate the tumour are rendered less effective by down regulation of MHC molecules on the APCs or tumour cells,^27^, ^28^ with less antigen presentation. As the tumour shifts the cytokine profile to one of immunosuppression, largely through signal transducer and activation of transcription (STAT)‐3 and nuclear factor‐kappa B (NF‐kB) signalling pathways,^29^ lymphocytes are differentiated to T‐regulatory cells (Tregs), rather than cytotoxic cells. Tregs themselves also deprive effector T cells of crucial survival and proliferation inducing cytokines which leads to their eventual demise.^30^ Finally, without the usual confines of growth restricting homeostasis, tumour growth out‐strips existing blood supply and neo‐vasculature develops. This is often leaky and fails to keep up with rapid growth leading to hypoxia in areas of the tumour, resulting in further genomic instability.^31^ Expression of hypoxia induced factors (HIFs) is increased, adding to the overall immunosuppressive milieu. Hypoxia leads to cytotoxic T cell exhaustion and increased Treg recruitment via HIFs,^32^ also upregulated by NF‐kB and STAT3 signalling.^33^

The importance of the TME has been shown in analysis of lung cancer samples. In a study examining the characteristics of lung cancer TME by whole exome and RNA‐seq analysis, Shinohara and colleagues classified tumours according to their degree of tumour proliferation, immunosuppression and anti‐tumour immunity.^34^ They showed clear associations between level of tumour proliferation and immunosuppression and poor clinical outcome, with immunosuppression perhaps being most important in outcomes, particularly in those tumours with high rates of progression.

**FIGURE 2:**
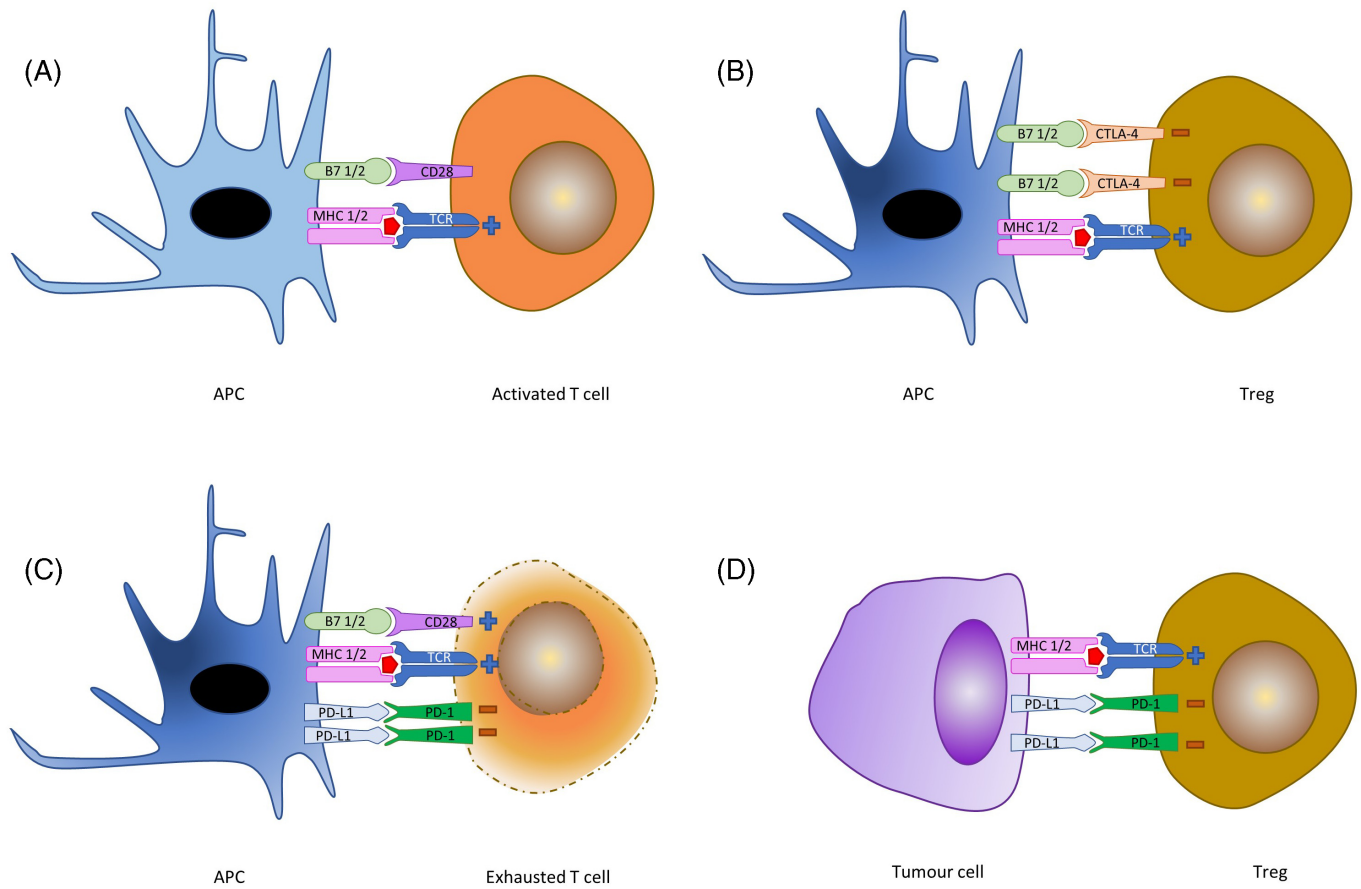
(A) Engagement of the TCR with an MHC molecule displaying a TAA on mature dendritic cells in the presence of CD28/B7 binding leads to T activation and proliferation, under the influence of proliferative cytokines including IL‐2 and IL‐4. TCR/MCH binding alone is inadequate to complete T cell activation and leads to reduced proliferation and poor effector function. (B) In contrast, if CTLA‐4 binds with B7 instead of CD28, the T cells are differentiated to Tregs, which directly inhibit CD8+ effector T cells through cytokine deprivation. (C) Immature DCs will express PD‐L1 binding to PD‐1 on T cells which have been activated over an extended period, which leads to T cell exhaustion. In addition to expression of PD‐L1, immature dendritic cells secrete negatively regulatory cytokines including TGFβ and IL‐10, further expanding the Treg pool and suppressing CD8+ cells. (D) Tumour cells are able to inducibly express PD‐L1 which promotes differentiation to Tregs and maintenance of the pro‐tumour milieu. Based on data from References 7, 30, 48. CLTA‐4, cytotoxic t‐lymphocyte associated protein‐4; MHC, major histocompatibility complex; PD‐1, programmed death‐1; PD‐L1, programmed death ligand‐1; TAA, tumour associated antigen; TCR, T cell receptor.

Immune checkpoints act to maintain homeostasis, avoid run away inflammation, and sustain self‐tolerance.^35^ They are part of the larger group of co‐stimulatory molecules essential in the modulation of the activity of inflammatory and immune cells. Their importance was demonstrated with the development of knock‐out mouse models, showing profound and fatal multi‐organ inflammation in mice lacking cytotoxic T‐lymphocyte associated protein‐4 (CTLA‐4).^36^ While there are many identified immune checkpoint proteins, CTLA‐4 and the broader B7‐CD28/CTLA‐4 superfamily is the best characterized. This co‐stimulatory pathway is critical in activation of T lymphocytes and development of T cell tolerance.^37^ While antigen presentation on MHC molecules to T‐cell receptors (TCRs) provides response specificity, it is insufficient to produce an activation response; the two step process.^38^ Activation of the T‐cell requires interaction of B7‐1 (CD80) or B7‐2 (CD86) on the APC, with the corresponding receptor on the T‐cell; CD28 (see Figure 2). The B7 molecules are exclusively present on the cell membranes of specialist APCs, and CD28 on T cells. Both TCR engagement with the MHC, and CD28 binding with its ligand are required for activation, with CD28 both lowering the threshold for stimulation and sustaining this activation.^39^ This interaction occurs early during T cell activation. CTLA‐4 is a CD28 homologue with higher affinity for the B7 ligand, but with a negative regulatory effect. The balance of CD28 to CTLA‐4 determines the resultant message. While CD28 is constitutively expressed on T cell membranes, CTLA‐4 is expressed only at a low level at baseline, with expression strongly induced following T‐cell activation, competing with CD28 for B7 ligands and acting to interfere with TCR function and downstream signalling pathways,^40^ providing a brake on immune activation. Interestingly, unlike effector T cells, Tregs have constitutively expressed CLTA‐4 which contributes to their immunosuppressive effects. In 1996, Allison and colleagues showed that antibodies directed against CTLA‐4 lead to rejection of existing tumours in a mouse model,^41^ and that this response remains in immunological memory on re‐challenge, suggesting a role for CTLA‐4 in tumour immune evasion.

Another potent checkpoint dimer in the B7‐CD28/CTLA‐4 superfamily is programmed death (PD)‐1 and its ligands, programmed death ligand (PD‐L)1 and PD‐L2, homologues of B7. As its names suggests it was initially thought to be responsible for apoptosis but was later found to be integral in immune regulation.^42^ Unlike CD28/CTLA‐4, expression of PD‐1 occurs across a number of cell types, including T and B cells, DCs and monocytes, in their activated state.^43^ Similarly, the primary ligand for PD‐1, PD‐L1, is found more broadly across immune cells, as well as non‐immune tissues including heart, pancreas, lung, liver and blood vessels,^44^ and is inducibly expressed on tumour cells.^45^ Less is known about the function of PD‐L2 which is thought to have a more restricted expression.^46^ While CTLA‐4 deficient mice died through unrestricted inflammation, Nishimura and colleagues described the development of autoimmune disease in PD‐1 deficient mice.^47^ This difference can be explained in part by the different roles the checkpoints play. While CTLA‐4 is important in the priming phase of T cell activation pathway and predominantly within lymphoid tissue, PD‐1's role is later in the activation cascade and exists in peripheral tissues.^48^ On binding with its ligand, PD‐1 diminishes the activation of effector T cells, promoting tolerance,^45^ and leading to T cell exhaustion. Iwai showed that PD‐1 deficient mice rejected the growth of injected myeloma cells, and injection of anti‐PD‐L1 into mice with established tumours lead to complete cure of the tumour,^49^ confirming a role for PD‐1/PD‐L1 in immune evasion by tumours. Blockade of PD‐1 was shown to enhance T cell mediated tumour cell cytolysis,^49^ and this effect has been shown to be most effective with intact CD28 signalling,^50^ giving a substantive rationale to CLTA‐4/PD‐1 dual blockade. Interestingly, in addition to the enhancement of cytolysis, PD‐1 blockade has been shown to reduced metastatic spread of tumours, at least in a mouse model.^51^

Both anti‐CLTA‐4 and anti‐PD‐1/anti‐PD‐L1 were tested across numerous animal studies of malignancy with variable results. In a mouse model of lung cancer, a homologue of ipilimumab blocking CTLA‐4 was ineffective as monotherapy but highly effective in combination with chemotherapy.^52^ In contrast, other soft‐tissue tumours considered to be immunogenic including bladder, fibrosarcoma and ovary, showed a complete response, while melanoma showed a poor response.^53^ However, it is evident that while there are significant parallels that can be drawn between murine and human immunology, disparate results were seen on a move into clinical trials. As an example, despite the disappointing results in murine models, human trials of Ipilimumab in advanced melanoma showed profound effects, at least for some individuals.^1^ In addition to the early work showing control of myeloma cells and colon cancer, PD‐1 blockade was also studied in combination with other immune therapies.^54^ Strome et al. showed that adoptive T‐cell therapy was substantially more effective at curing squamous cell carcinoma in mice when combined with PD‐L1 blockade.^55^ Similarly, blockade of PD‐1 or PD‐L1 along with a cellular vaccine was more effective at killing melanoma cells than the vaccine alone.^56^

The mechanism of immune‐related adverse effects is poorly understood. Genetic loss of function polymorphisms of CTLA‐4 has been studied and an associated auto‐immune phenotype described,^57^ so the implications of blocking immune checkpoints were known. Unfortunately, while mouse models of CTLA‐4/PD‐1 knock‐out showed substantial immune dysregulation, irAEs were not a feature of pre‐clinical studies.^58^ There have been efforts to develop appropriate mouse models to study irAEs, but these have been limited in scope.^59^ Overall, adverse effects are seen more commonly in CTLA‐4 blockade, perhaps related to the early effect on priming, although some adverse effects are seen more commonly with anti‐PD‐1/PD‐L1 therapies including pneumonitis.^60^ Unsurprisingly, along with the improvement in clinical response, dual checkpoint blockade is associated with higher rates of irAEs.^61^

While use of checkpoint inhibitors in routine clinical practice is relatively recent, the science behind them is relatively ‘old’. This commentary has highlighted the value of extensive basic science of T cell immunobiology and how it has yielded insights that combined with preclinical cancer models provided an exceptional example of the power of translational science to design novel cancer treatments.
